# Efficacy of a novel one-step knife compared to conventional knife for colorectal endoscopic submucosal dissection: a prospective multicenter randomized controlled trial

**DOI:** 10.1007/s00384-025-04910-0

**Published:** 2025-05-14

**Authors:** Hong Jun Park, Su Young Kim, Gwang Ho Baik, Myeongsook Seo, Hyun Il Seo, Sung Chul Park, Hyunil Kim, Hyun-Soo Kim

**Affiliations:** 1 Division of Gastroenterology, Department of Internal Medicine, Yonsei University Wonju College of Medicine, 20 Ilsan-Ro, Wonju, 26426 Republic of Korea; 2 Department of Internal Medicine, Hallym University College of Medicine, Chuncheon, Korea; 3 Department of Gastroenterology, Gangneung Asan Hospital, University of Ulsan College of Medicine, Gangneung, Republic of Korea; 4 Department of Internal Medicine, Kangwon National University College of Medicine, Chuncheon, Republic of Korea

**Keywords:** Endoscopic submucosal dissection, Colorectal neoplasm, Colonoscopy, Endoscopic knife

## Abstract

**Purpose:**

For the treatment of advanced colorectal neoplasms, colon endoscopic submucosal dissection (ESD) is a crucial technique, although it is time-consuming. The purpose of this study was to evaluate the efficacy of a recently developed one-step knife (OSK) in colon ESD and compare its performance with that of a conventional knife (CK).

**Methods:**

Between July 2020 and November 2021, patients scheduled to undergo colorectal ESD were randomly assigned to either the OSK group or the CK group. The primary outcome was the total submucosal injection time. Additionally, total procedure time, treatment outcomes, adverse events, and operator convenience were analyzed.

**Results:**

Data from 53 patients (28 in the OSK group and 25 in the CK group) were analyzed. The mean total injection time was lower in the OSK group than in the CK group (186 s [IQR, 116.8–249.5] vs. 265 s [IQR, 130.5–553.0]), but the difference was not statistically significant (*P* = 0.082). The total procedure time tended to be shorter in the OSK group than in the CK group (15.5 min [IQR, 11.3–22.8] vs. 20 min [IQR, 13.5–42.5], *P* = 0.110). Resection rates and adverse events did not differ between the two groups. A greater proportion of endoscopists expressed high satisfaction with the OSK, particularly regarding submucosal injection.

**Conclusion:**

Compared to the CK, OSK use led to shorter injection and procedure times, though not statistically significant. The use of this newly developed endoscopic knife can potentially enhance the effectiveness and efficiency of colorectal ESD (Clinical Research Information Service: KCT0005123).

**Supplementary information:**

The online version contains supplementary material available at 10.1007/s00384-025-04910-0.

## Introduction

Colorectal endoscopic submucosal dissection (ESD) is a cornerstone in the management of colorectal tumors, including early colorectal cancer (CRC) [1, 2]. ESD enables en bloc resection irrespective of tumor size, offering significant advantages such as a high curative resection rate and a remarkably low local recurrence rate, especially when compared to endoscopic mucosal resection (EMR) [1, 3, 4]. Despite its benefits, ESD is associated with certain limitations, including a steep learning curve, extended procedure times, and a higher risk of complications relative to EMR [1, 5]. In particular, prolonged procedure durations can increase patient discomfort and elevate the risk of procedure-related complications [6–8].

**Fig. 1:**
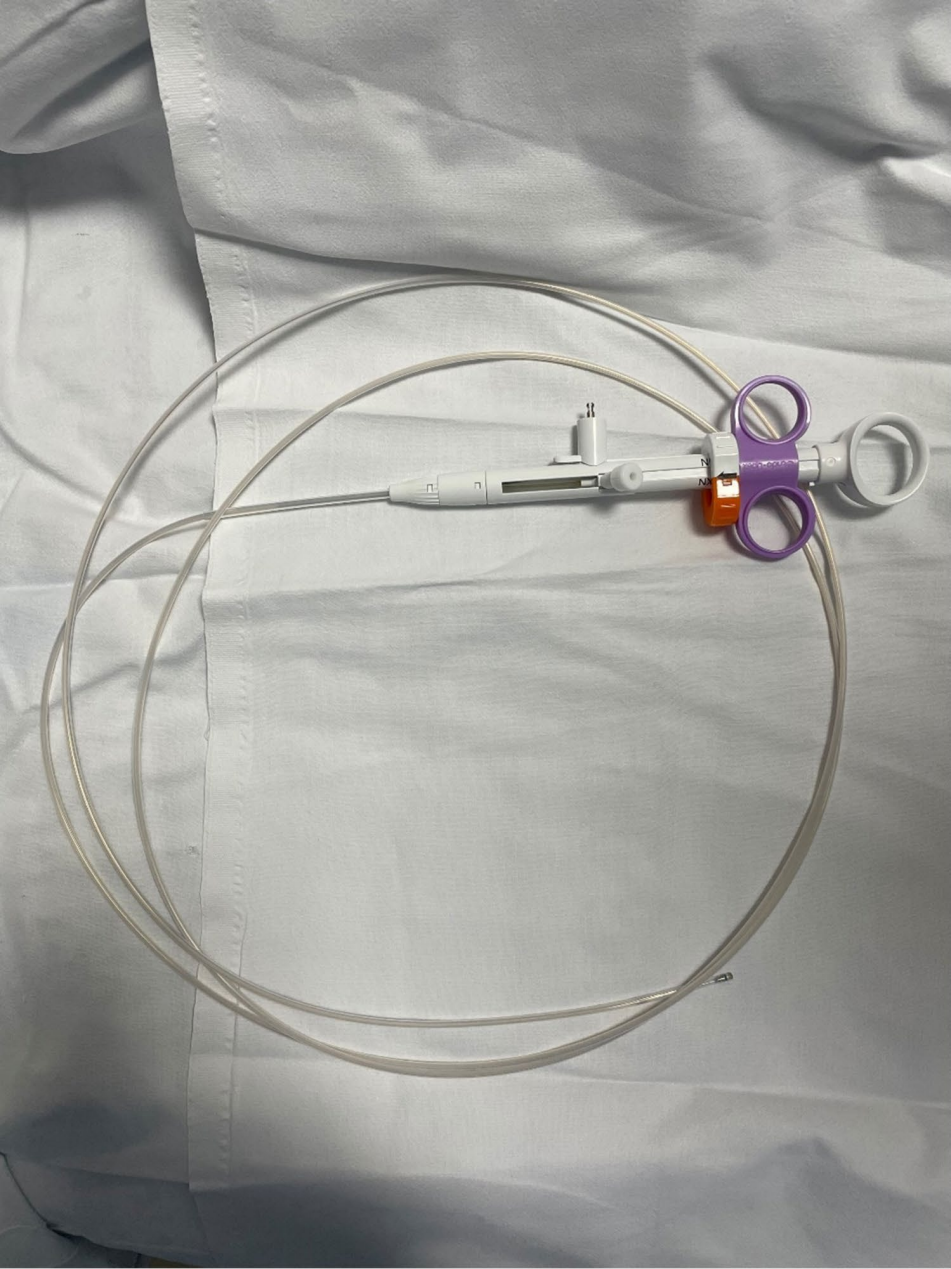
Endo-Upex™ Onestep ESD Knife

ESD is a multi-step procedure that involves thermal marking of the lesion, submucosal injection, circumferential mucosal incision, and submucosal dissection. Each stage requires a distinct endoscopic device introduced through the endoscopic channel. Among these steps, submucosal injection is particularly critical, as it helps prevent perforation during the mucosal incision and creates a dissection plane within the submucosa. Typically, a single ESD procedure necessitates more than two submucosal injections. However, this step is labor-intensive and time-consuming, involving multiple sequential actions such as withdrawing the endoscopic knife, inserting the needle injector, delivering fluid into the submucosa, removing the needle injector, and reinserting the endoscopic knife. Each submucosal injection takes over a minute to perform, and the need for multiple injections can considerably prolong the overall procedure time. To address this challenge, our research team recently developed a novel one-step knife (OSK) and conducted preclinical testing in animal models. Following the preclinical phase, we conducted a clinical study focusing on gastric ESD, the results of which have been published [9]. The OSK is designed with a single channel that allows alternating use of the endoscopic knife and needle injector (Fig. 1). This design significantly reduces the time required to switch between the injector and knife during submucosal injection, thereby enhancing procedural convenience. To date, no clinical studies have evaluated the use of a OSK in colorectal ESD. Therefore, we conducted a multicenter randomized controlled trial (RCT) to compare the efficacy and safety of the OSK with those of the conventional dual knife in colorectal ESD.

## Methods

### Patients and study design

This study was a multicenter, single-blind, RCT conducted on patients scheduled for colorectal ESD. Participants aged ≥ 19 years with colorectal neoplasms requiring ESD were recruited from four tertiary care hospitals between July 2020 and November 2021. Eligible patients were randomly assigned to the OSK group (1.5-mm Endo-UpexTM Onestep ESD Knife; Upexmed, Anyang-si, Republic of Korea) or the conventional knife (CK) group (dual knife; DualKnifeTM, Olympus, Tokyo, Japan). We excluded patients with a history of colorectomy or severe comorbidities, and those who refused to participate. Patients were also excluded if surgery was deemed necessary instead of ESD for their colorectal lesions or if an additional knife other than the assigned one was used during the procedure. The study protocol was approved by the Institutional Review Board of Wonju Severance Christian Hospital (approval no. CR220001). The study was conducted in accordance with the Declaration of Helsinki. Written informed consent was obtained from all participants prior to enrollment. The trial was registered with the Clinical Research Information Service (KCT0005123).

**Fig. 2:**
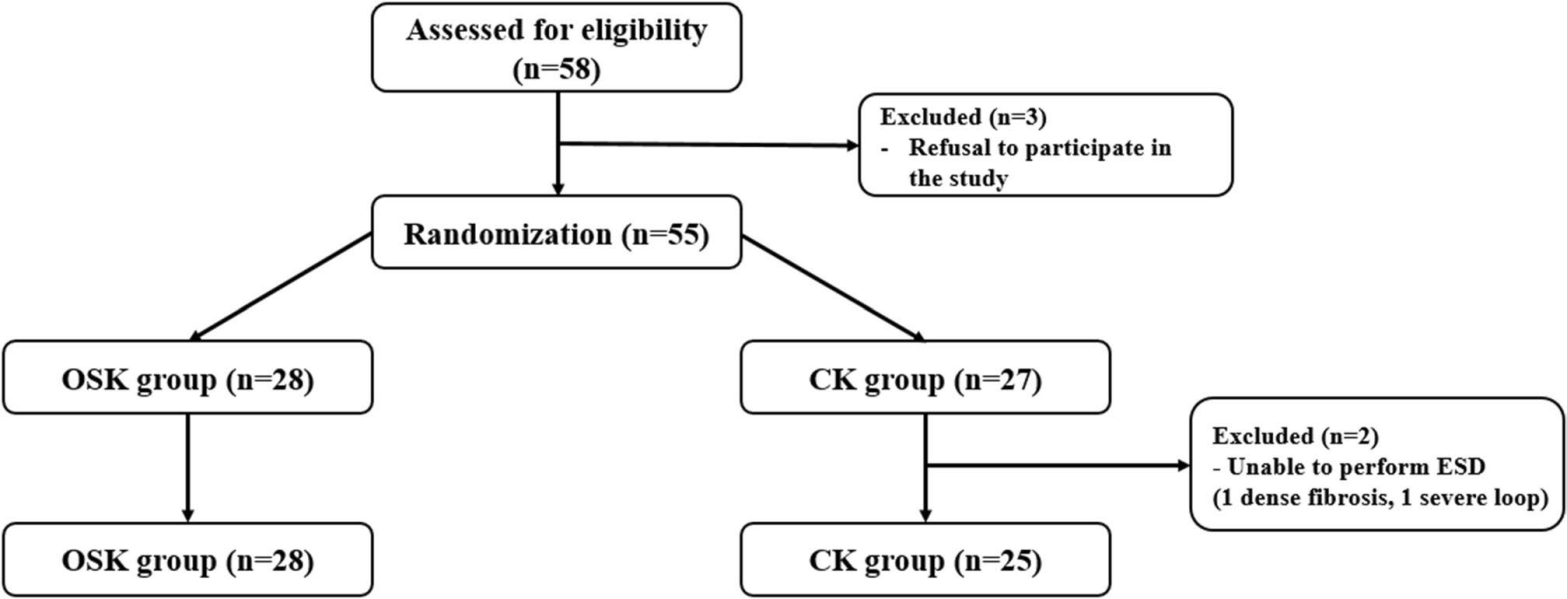
Flow diagram of this study. OSK, one-step knife; CK, conventional knife; ESD, endoscopic submucosal dissection

During the study period, 58 patients were assessed for enrollment. After excluding 3 patients who withdrew from the study, 55 patients were randomly assigned to either the OSK group or the CK group. Randomization was performed using a computer-generated random number table, ensuring a 1:1 distribution of participants. In the CK group, two patients were excluded from the final analysis: one required surgery due to severe fibrosis, which rendered ESD unfeasible, and the other underwent surgery because endoscopic access to the lesion was not possible. Ultimately, the final analysis included 28 patients from the OSK group and 25 patients from the CK group (Fig. 2).

### ESD procedure

All procedures were performed by six expert endoscopists, each with experience in more than 100 ESD cases. The endoscopic procedures utilized standard colonoscopes (CF-HQ290L, CF-HQ290I, or PCF-H290 TI; Olympus, Tokyo, Japan) fitted with a transparent tip hood. The lesion targeted for ESD was initially identified using white-light endoscopy and narrow-band imaging to precisely delineate its borders. Submucosal injection was then performed in both groups using a solution of saline mixed with epinephrine (0.01 mg/mL) and 0.8% indigo carmine to achieve adequate lesion lifting. After sufficient lifting was confirmed, mucosal incision and submucosal dissection were performed. In the OSK group, the injection needle was integrated into the OSK, eliminating the need to insert an additional needle injector through the endoscopic channel for submucosal injection. Transitioning between the knife and needle was performed seamlessly by manipulating the knife handle. Submucosal injection, mucosal incision, and submucosal dissection were successfully completed using only the OSK, eliminating the need for an additional injection needle. Conversely, the CK group required the use of a separate needle injector for submucosal injection. After completing the injection, the injector was removed from the endoscopic channel, and the dual knife was reinserted to proceed with the incision and dissection. A high-frequency generator (VIO300D; ERBE, Tübingen, Germany) was used during ESD. Post-procedure, chest and abdominal X-rays were performed to assess for adverse events such as perforation.

### Outcomes and definitions

The primary outcome of the study was the submucosal injection time. Secondary outcomes included the total procedure time, treatment outcomes, adverse events, and operator convenience.

In accordance with a previous study [9], the submucosal injection time in the CK group was defined as the interval between the withdrawal of the endoscopic knife for submucosal injection and its reinsertion. In the OSK group, it was defined as the interval between the switchover from knife to injector for submucosal injection and the switchover back to the knife. The total procedure time was measured as the interval between the first marking and specimen detachment. To ensure objectivity and accuracy in measuring time-related outcomes, the submucosal injection time, total procedure time, and the number of injections were recorded in real time by a research nurse observing the procedure, rather than by the operator.

Patient data including age, sex, body mass index, comorbidities, previous abdominal surgery, and use of antithrombotic agents were collected. Additionally, endoscopy-related parameters such as bowel preparation quality, tumor location, and tumor morphology were recorded. For histopathologic evaluation, data on lesion size, tumor involvement, depth of invasion, and the presence of lymphovascular invasion were obtained by reviewing histology slides and pathology reports. Furthermore, endoscopists and assistants completed a device satisfaction questionnaire. This survey included questions about overall satisfaction, satisfaction with the procedural process (whether the knife functions well during incision and dissection), and satisfaction with submucosal injection. Responses were measured on a 5-point scale (very satisfactory, satisfactory, somewhat satisfactory, unsatisfactory, and very unsatisfactory) (Supplementary Table 1).

Adverse events included bleeding and perforation. Intra-procedural bleeding was defined as significant active bleeding that required the use of additional devices, such as Coagrasper Hemostatic Grasper (Single Use [FD-410LR], Olympus, Tokyo, Japan). Delayed bleeding was defined as active bleeding occurring from the completion of the procedure up to 1 week post-procedure. Perforation was defined as either endoscopically identified during the procedure or as the presence of free air detected on chest or abdominal radiographs following the procedure.

### Statistical analysis

We hypothesized that the OSK would reduce the submucosal injection time by minimizing the time required for device insertion and withdrawal during colon ESD. Based on previous animal and clinical studies [9], we estimated the average injection time to be 50 s in the OSK group and 90 s in the CK group, with a standard deviation of 25 s. The statistical power was set at 80%, with a significance level of 0.05, necessitating at least 24 participants per group. Accounting for a 15% dropout rate, 56 participants were required, with 28 individuals assigned to each group.

Associations between categorical variables were assessed using the chi-square test or Fisher’s exact test, whereas the Mann–Whitney *U* test was used for other variables. All *p*-values were two-sided and considered statistically significant if *P* < 0.05. Statistical analyses were conducted using SPSS software (version 26.0; IBM Corp., Armonk, NY, USA).

## Results

### Baseline characteristics of patients and colorectal lesions

Table 1 presents the baseline characteristics of the study population and the colonoscopy findings. No significant differences were observed between the groups in terms of age, sex, body mass index, underlying comorbidities, previous abdominal surgery, use of antithrombotic agents, or bowel preparation quality. Table 2 presents the clinicopathological characteristics of the resected colorectal lesions. Histopathological diagnoses revealed that adenomas were the most common finding in both groups, with the rectum being the most frequent tumor location in both groups. No significant differences were found between the groups in terms of histopathologic diagnosis or tumor location. Laterally spreading tumors were the most common tumor morphology in both groups. Additionally, the size of the resected specimen and the lesion size showed no significant differences between the two groups.

**Table 1:** Baseline characteristics of the study population and colonoscopy

Variables	OSK group (*n* = 28)	CK group (*n* = 25)	*P* value
Age, mean ± SD, years	65.1 ± 9.3	65.9 ± 11.1	0.794
Sex, male, *n* (%)	19 (67.9%)	14 (56.0%)	0.374
BMI, mean ± SD	24.4 ± 2.8	24.5 ± 4.1	0.912
Comorbidities, *n* (%)			
Hypertension	16 (57.1%)	14 (56.0%)	0.933
Diabetes mellitus	8 (28.6%)	6 (24.0%)	0.706
Cardiovascular disease	1 (3.6%)	4 (16.0%)	0.176
Cerebrovascular disease	4 (14.3%)	1 (4.0%)	0.355
Chronic kidney disease	0	1 (4.0%)	0.472
Liver cirrhosis	0	1 (4.0%)	0.472
Pulmonary disease	2 (7.1%)	3 (12.0%)	0.658
Previous abdominal surgery history, *n* (%)	2 (7.1%)	2 (8.0%)	0.860
Use of antithrombotic agents, *n* (%)	2 (7.1%)	3 (12.0%)	0.393
Bowel preparation, *n* (%)			
Right colon			0.163
Poor	0	1 (4.0%)	
Good	10 (35.7%)	4 (16.0%)	
Excellent	18 (64.3%)	20 (80.0%)	
Transverse colon			0.081
Poor	0	1 (4.0%)	
Good	8 (28.6%)	2 (8.0%)	
Excellent	20 (71.4%)	22 (88.0%)	
Left colon			0.851
Poor	1 (3.6%)	1 (4.0%)	
Good	5 (17.9%)	3 (12.0%)	
Excellent	22 (78.5%)	21 (84.0%)	

*OSK* one-step knife, *CK* conventional knife, *SD* standard deviation, *BMI* body mass index

**Table 2:** Clinicopathological characteristics of resected colorectal lesions

Variables	OSK group (*n* = 28)	CK group (*n* = 25)	*P* value
Histopathologic diagnosis, *n* (%)			0.997
Adenoma	10 (35.8%)	10 (40.0%)	
Adenoma with HGD	7 (25.0%)	6 (24.0%)	
Intramucosal adenocarcinoma	6 (21.4%)	5 (20.0%)	
Adenocarcinoma with WD	1 (3.6%)	1 (4.0%)	
Adenocarcinoma with MD	2 (7.1%)	1 (4.0%)	
NET	2 (7.1%)	2 (8.0%)	
Tumor location, *n* (%)			0.930
Ascending colon	6 (21.4%)	8 (32.0%)	
Hepatic flexure	1 (3.6%)	1 (4.0%)	
Transverse colon	4 (14.3%)	3 (12.0%)	
Descending colon	3 (10.7%)	2 (8.0%)	
Sigmoid colon	1 (3.6%)	2 (8.0%)	
Rectum	13 (46.4%)	9 (36.0%)	
Tumor morphology			0.857
Non-LST	5 (17.9%)	4 (16%)	
LST	23 (82.1%)	21 (84%)	
Homogenous	3	3	
Nodular mixed	10	7	
Flat elevated	9	8	
Pseudodepressed	1	3	
Size of resected specimen, median [IQR], mm	22 [17.3–30.0]	24 [17.0–32.5]	0.681
Size of lesion, median [IQR], mm	18.5 [15.0–25.0]	20 [13.0–27.0]	0.929

*OSK* one-step knife, *CK* conventional knife, *HGD* high-grade dysplasia, *WD* well-differentiated, *MD* moderately differentiated, *NET* neuroendocrine tumor, *LST* laterally spreading tumor, *IQR* interquartile range

### Efficacy assessment

The total median procedure time was 15.5 min (interquartile range [IQR], 11.3–22.8) in the OSK group and 20 min (IQR, 13.5–42.5) in the CK group (*P* = 0.110). The median time required for total submucosal injection was 186 s (IQR, 116.8–249.5) in the OSK group and 265 s (IQR, 130.5–553.0) in the CK group (*P* = 0.082). The median number of total injections was the same in both groups (Table 3). The rates of en bloc resection, R0 resection, and curative resection showed no statistically significant differences between the two groups (96.4% vs. 96.0%, *P* = 0.935; 96.4% vs. 84.0%, *P* = 0.122; 89.3% vs. 84.0%, *P* = 0.570, respectively). Seven patients did not achieve curative resection for the following reasons: in two patients, the lesions were macroscopically completely removed but involved piecemeal resection of some tissues; in three patients, horizontal margin positivity (focal extension of adenoma) was observed; and in two patients, lymphatic invasion or submucosal invasion depth exceeding 1000 µm was detected.

**Table 3:** Procedure-related characteristics in both groups

Variables	OSK group (*n* = 28)	CK group (*n* = 25)	*P* value
Total procedure time, median [IRQ], min	15.5 [11.3–22.8]	20 [13.5–42.5]	0.110
Total submucosal injection time, median [IQR], sec	186 [116.8–249.5]	265 [130.5–553.0]	0.082
Total injection number, median [IQR]	4 [3–6]	4 [3–7]	0.935
Total injection amount, median [IQR], mL	23.5 [13.5–34.8]	21.0 [15.0–54.5]	0.643
En bloc resection	27 (96.4%)	24 (96.0%)	0.935
R0 resection	27 (96.4%)	21 (84.0%)	0.122
Curative resection	25 (89.3%)	21 (84.0%)	0.570
Adverse events			
Intra-procedural bleeding	2 (7.1%)	4 (16.0%)	0.404
Delayed bleeding	2 (7.1%)	0	0.492
Perforation	0	0	-

*OSK* one-step knife, *CK* conventional knife, *IQR* interquartile range

### Safety and satisfaction assessment

**Fig. 3:**
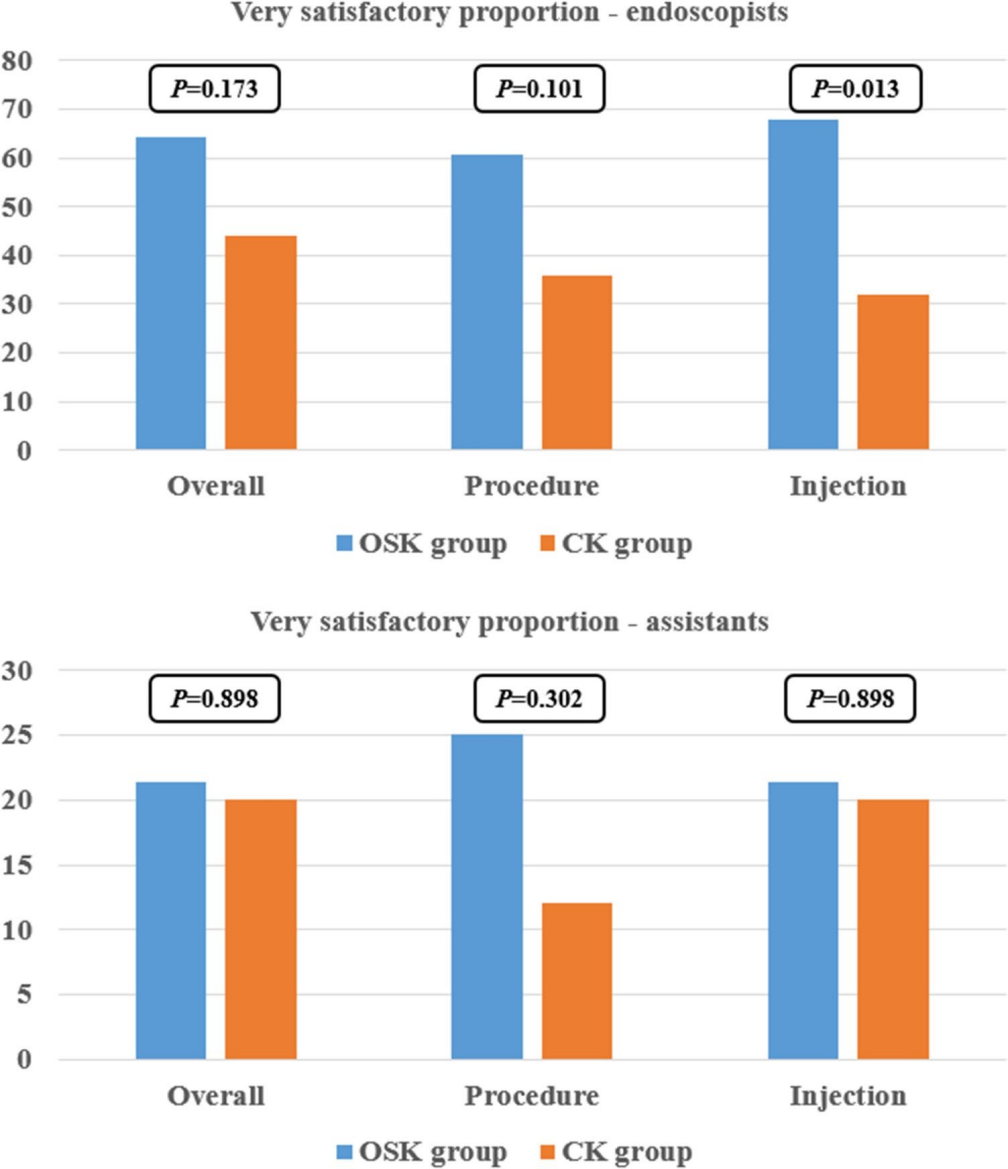
Analysis of levels of contentment with regard to endoscopic knives. OSK, one-step knife; CK, conventional knife

Intra-procedural bleeding was confirmed in two patients (7.1%) in the OSK group and four patients (16.0%) in the CK group (*P* = 0.404). Delayed bleeding occurred in two patients (7.1%) in the OSK group only. No cases of perforation were observed in either group (Table 3). All instances of bleeding were successfully managed endoscopically, and no severe complications were reported in either group. Regarding the questionnaire responses, endoscopists showed a tendency toward higher satisfaction with the device compared to assistants. Notably, endoscopists provided a statistically significant positive response, indicating that they found the OSK very satisfactory for submucosal injection (*P* = 0.013) (Fig. 3).

### Subgroup analyses of total submucosal time

The median total submucosal injection time in the proximal colon was significantly lower in the OSK group compared to the CK group (138.0 [105.0–244.0] s vs. 339.0 [132.5–707.3] s, respectively; *P* = 0.037). However, no significant differences were observed between the groups in the injection time in the distal colon (Table 4).

**Table 4:** Results of the subgroup analysis of total submucosal time related to location and resected specimen size between the two groups

Median [IQR], sec	OSK group (*n* = 28)	CK group (*n* = 25)	*P* value
Location			
Proximal colon^a^	138.0 [105.0–244.0]	339.0 [132.5–707.3]	0.037
Distal colon^b^	196.0 [147.0–280.0]	234.0 [123.5–341.0]	0.773
Size			
≥ 25 mm	258.5 [174.8–416.3]	421.5 [253.8–846.3]	0.123
< 25 mm	147.0 [106.0–202.8]	162.0 [97.5–339.0]	0.650

*OSK* one-step knife, *CK* conventional knife, *IQR* interquartile range

^a^The proximal colon refers to the combined regions of the cecum, ascending colon, and transverse colon

^b^The distal colon refers to the combined regions of the descending colon, sigmoid colon, and rectum

## Discussion

The is the first multicenter, prospective RCT worldwide to evaluate the efficacy of a novel OSK in colorectal ESD. The results showed a reduction in total submucosal injection time in the OSK group compared to the CK group, although the difference was not statistically significant. Notably, this difference was more pronounced in the proximal colon. Additionally, endoscopists’ satisfaction with submucosal injection was significantly higher in the OSK group than in the CK group. There were no significant group differences in curative resection rates or adverse events. These findings suggest that the OSK offers improved efficacy and greater therapeutic comfort compared to the CK in colorectal ESD.

ESD is a time-intensive procedure comprising multiple steps, among which submucosal injection is a crucial stage. Conventionally, submucosal injection requires device changes during the procedure. The newly developed OSK integrates both a knife and a needle injector into a single device, eliminating the need for device exchange, thereby reducing time consumption and enhancing convenience for endoscopists. In our study, the OSK group demonstrated a reduction in total submucosal injection time compared to the CK group, although this difference did not reach statistical significance. The lack of statistical significance may be due to the relatively short overall procedure time in this study. Compared to previous studies utilizing other endoscopic knives and devices [10–13], the total procedure time in our study was relatively short, and may have been insufficient to achieve statistical significance.

Interestingly, subgroup analysis revealed that when ESD was performed in the proximal colon, the total submucosal injection time was significantly reduced in the OSK group compared to the CK group. This finding can be attributed to several factors. First, in the proximal colon, the tumor’s position can shift easily due to the patient’s breathing patterns. When devices are exchanged through the endoscopy channel, these positional changes often require additional time to readjust the tumor’s location in the endoscopic view. Conversely, the OSK eliminates the need for device exchange, allowing seamless transitions between the knife and injector while maintaining a fixed position, thus minimizing the impact of respiratory movements. Second, the endoscope in the proximal colon is sometimes not straight, and loops may form during the procedure. This can create difficulties and resistance when exchanging devices through the endoscopic channel. Therefore, when performing ESD on lesions in the proximal colon, the use of an OSK offers the advantage of reduced total submucosal injection time, thereby facilitating a more efficient and streamlined procedure.

To date, various types of knives have been developed to enhance the convenience of colon ESD procedures [12, 14–17]. However, our study is the first prospective RCT to evaluate a knife that integrates an injector, enabling seamless incision and injection without the need for device changes. Recently, a hybrid knife suitable for colon ESD has been developed, which is functionally similar to the OSK used in this study. However, clinical studies on the hybrid knife have been limited to retrospective analyses, with no prospective studies being available, making it challenging to fully assess the effectiveness [13]. Moreover, the previously developed hybrid knife employs high-pressure water jet injection for submucosal injection. While this method is generally considered safe, there are concerns about the potential for microperforation caused by high-pressure injection, particularly in cases with severe fibrosis or thin mucosal layers. Conversely, the newly developed OSK incorporates a conventional injector within the knife, allowing submucosal injection to be performed using standard techniques and thereby eliminating these concerns.

Complete resection of colorectal tumors during ESD is crucial, as it is closely associated with both the treatment outcomes and the prevention of future recurrence [18–20]. In this study, there were no significant differences between the OSK and CK groups in terms of the en bloc resection, R0 resection, or curative resection rates. Similarly, no significant differences in adverse events were observed between the two groups in this RCT, confirming the efficacy of the newly developed OSK for colon ESD. Additionally, with regard to submucosal injection, endoscopists reported that the satisfaction rate in the OSK group was more than double that in the CK group. This satisfaction is likely attributable to the ability of the OSK to bypass cumbersome steps during submucosal injection and allow for a seamless transition between incision and dissection.

Despite the intriguing findings of our study, several limitations should be considered. First, although our RCT successfully analyzed data from 53 patients, the sample size in each group was relatively small, precluding comprehensive evaluation of the secondary outcomes. Second, considering that operator blinding to the knife type used was not feasible, subjective factors may have influenced the assessment of the knife’s convenience.

In conclusion, this is the first RCT to compare the efficacy of the OSK and CK in colon ESD. The use of the OSK in colon ESD was found to be safe, convenient for the operator, and efficient. The OSK demonstrated a tendency to reduce the submucosal injection time, with a particularly pronounced reduction observed during ESD of lesions located in the proximal colon. The newly developed OSK is expected to facilitate more effective performance of the technically demanding colon ESD.

## Supplementary information

Below is the link to the electronic supplementary material.

ESM 1 (DOCX 15.3 KB)

## Data Availability

The data generated and analyzed during this study are available from the corresponding author upon reasonable request.
