# Supplementary material for: Efficacy of a novel one-step knife compared to conventional knife for colorectal endoscopic submucosal dissection: a prospective multicenter randomized controlled trial
Source: Int J Colorectal Dis. 2025 May 14;40(1):116. doi: 10.1007/s00384-025-04910-0 (PMC12078380; doi:10.1007/s00384-025-04910-0)
Supplement: Supplementary file 1 — (DOCX 15.3 KB) [file 384_2025_4910_MOESM1_ESM.docx]

**Supplementary table 1. Satisfaction with endoscopic knives in both groups**

| Overall | OSK group  (n = 28) | CK group  (n = 25) | *P* value |
| --- | --- | --- | --- |
| Endoscopists  Very satisfactory  Satisfactory  Neutral  Unsatisfactory  Very Unsatisfactory | 18 (64.3%)  9 (32.1%)  1 (3.6%)  0  0 | 11 (44.0%)  9 (36.0%)  4 (16.0%)  1 (4.0%)  0 | 0.228 |
| Assistants  Very satisfactory  Satisfactory  Neutral  Unsatisfactory  Very Unsatisfactory | 6 (21.4%)  16 (57.2%)  5 (17.8%)  1 (3.6%)  0 | 5 (20.0%)  14 (56.0%)  5 (20.0%)  1 (4.0%)  0 | 0.997 |
| Procedure | OSK group  (n = 28) | CK group  (n = 25) | *P* value |
| Endoscopists  Very satisfactory  Satisfactory  Neutral  Unsatisfactory  Very Unsatisfactory | 17 (60.7%)  10 (35.7%)  1 (3.6%)  0  0 | 9 (36.0%)  11 (44.0%)  1 (4.0%)  4 (16.0%)  0 | 0.095 |
| Assistants  Very satisfactory  Satisfactory  Neutral  Unsatisfactory  Very Unsatisfactory | 7 (25.0%)  15 (53.6%)  5 (17.8%)  1 (3.6%)  0 | 3 (12.0%)  16 (64.0%)  5 (20.0%)  1 (4.0%)  0 | 0.690 |
| Injection | OSK group  (n = 28) | CK group  (n = 25) | *P* value |
| Endoscopists  Very satisfactory  Satisfactory  Neutral  Unsatisfactory  Very Unsatisfactory | 19 (67.8%)  8 (28.6%)  1 (3.6%)  0  0 | 8 (32.0%)  13 (52.0%)  3 (12.0%)  1 (4.0%)  0 | 0.057 |
| Assistants  Very satisfactory  Satisfactory  Neutral  Unsatisfactory  Very Unsatisfactory | 6 (21.4%)  15 (53.6%)  3 (10.7%)  4 (14.3%)  0 | 5 (20.0%)  15 (60.0%)  4 (16.0%)  1 (4.0%)  0 | 0.600 |

OSK, one-step knife; CK, conventional knife.
